# Pulse Wave Velocity as a Marker of Vascular Dysfunction and Its Correlation with Cardiac Disease in Children with End-Stage Renal Disease (ESRD)

**DOI:** 10.3390/diagnostics12010071

**Published:** 2021-12-29

**Authors:** Cristina Filip, Cătălin Cirstoveanu, Mihaela Bizubac, Elena Camelia Berghea, Andrei Căpitănescu, Mihaela Bălgrădean, Carmen Pavelescu, Alin Nicolescu, Marcela Daniela Ionescu

**Affiliations:** 1 Pediatric Cardiology, M.S. Curie Children’s Hospital, Constantin Brâncoveanu Boulevard, No. 20, 4th District, 041451 Bucharest, Romania; filipcristina06@yahoo.com (C.F.); nicolescu_a@yahoo.com (A.N.); 2 Neonatal Intensive Care Unit, M.S. Curie Children’s Hospital, Constantin Brâncoveanu Boulevard, No. 20, 4th District, 041451 Bucharest, Romania; anamihaeladogaru@yahoo.com; 3 Department of Neonatal Intensive Care, “Carol Davila” University of Medicine and Pharmacy, 020021 Bucharest, Romania; drmarcela.ionescu@gmail.com; 4 Department of Pediatrics, “Carol Davila” University of Medicine and Pharmacy, 020021 Bucharest, Romania; camelia.berghea@umfcd.ro (E.C.B.); mihaela.balgradean@umfcd.ro (M.B.); 5 Allergology and Clinical Immunology Department, M.S. Curie Children’s Hospital, Constantin Brâncoveanu Boulevard, No. 20, 4th District, 041451 Bucharest, Romania; 6 Pediatric Hemodialysis, M.S. Curie Children’s Hospital, Constantin Brâncoveanu Boulevard, No. 20, 4th District, 041451 Bucharest, Romania; andreicapitanescu@gmail.com; 7 Pediatrics and Pediatric Nephrology, M.S. Curie Children’s Hospital, Constantin Brâncoveanu Boulevard, No. 20, 4th District, 041451 Bucharest, Romania; carmen_pavelescu@yahoo.com; 8 Pediatrics and Pediatric Pulmonology, M.S. Curie Children’s Hospital, Constantin Brâncoveanu Boulevard, No. 20, 4th District, 041451 Bucharest, Romania

**Keywords:** pulse wave velocity, vascular dysfunction, cardiac disease, dialysis, kidney disease

## Abstract

One of the main markers of arterial stiffness is pulse wave velocity (PWV). This parameter is well studied as a marker for end-organ damage in the adult population, being considered a strong predictor of major cardiovascular events. This study assessed PWV in children with chronic kidney disease (CKD) as a marker of cardiovascular risk. We conducted a prospective observational single-center cohort study of 42 consecutively pediatric patients (9–18 years old) with terminal CKD and dialysis, at the Hemodialysis Department of the “M. S. Curie” Hospital, Bucharest. We measured PWV by echocardiography in the ascending aorta (AscAo) and the descending aorta (DescAo), and we correlated them with left ventricular hypertrophy (LVH). Fifteen patients (35.7%) presented vascular dysfunction defined as PWV above the 95th percentile of normal values in the AscAo and/or DescAo. Cardiac disease (LVH/LV remodeling) was discovered in 32 patients (76.2%). All patients with vascular damage also had cardiac disease. Cardiac damage was already present in all patients with vascular disease, and the DescAo is more frequently affected than the AscAo (86.6% vs. 46.9%). Elevated PWV could represent an important parameter for identifying children with CKD and high cardiovascular risk.

## 1. Introduction

The main leading cause of death in end-stage renal disease (ESRD) is cardiovascular disease (CVD) [1,2]. The presence of cardiomyopathy (left ventricle hypertrophy/remodeling), as well as early signs of atherosclerosis (increased values of the carotid artery intima-media thickness, arterial stiffness), are very often present in children with CKD, and especially in children who are treated with dialysis [3]. The measurement of PWV is considered the non-invasive gold standard method to assess the aortic stiffness and to evaluate the mechanical properties of large elastic arteries, but its measurement in children is challenging due to technical difficulties, the aspects related to growth and low standardization between algorithms for calculating PWV [4,5,6,7,8,9]. Recent research has focused on identifying the presence of early cardiovascular abnormalities in children with CKD. Left ventricular (LV) abnormalities such as LV hypertrophy (LVH) and LV dysfunction, damage to the large arteries such as arterial stiffness, increased intima-medial thickness (IMT) of the carotids, and coronary/carotid calcifications are now accepted as early markers of cardiomyopathy and atherosclerosis. These markers are strong, independent predictors of cardiac morbidity and mortality, in children with CKD [10]. CVD was described as the cause of death in children with CKD in 23% compared with 3% in the general pediatric population [11].

There is emerging literature describing arterial stiffness in pediatric populations and the relation of arterial stiffness to pediatric CKD, but this study has primarily focused on pediatric patients with terminal CKD and dialysis. Measuring the vascular rigidity/elasticity parameters and correlating them with cardiac disease markers (e.g., impaired cardiac function, ventricular hypertrophy, diastolic dysfunction) in CKD may offer an additional predictive value for major cardiovascular events and mortality [12,13,14,15].

According to the recommendations of the American Heart Association from 2015 [16], there are two methods to determine the distance covered by the pulse wave in the aorta for an adult patient and consecutively used to calculate the carotid-femoral PWV: the subtraction method (subtract the distance from the suprasternal notch to the carotid artery from the distance from the suprasternal notch to the femoral artery) and the direct measurement method (multiply the distance from the carotid artery to the femoral artery by 0.8). Many studies have used the magnetic resonance technique to precisely determine the aortic length [17,18,19,20], but this method implies high costs, risks, and specific contraindications. Furthermore, the pediatric cases present the additional disadvantage of the necessity for anesthesia during the examination.

Regarding the measurement of PWV in children, a method has not been validated yet, given the fact that this parameter is closely dependent on body dimensions and height. More recently, some research studies have evaluated the results of various calculation methods for PWV and tried to determine which one is less prone to errors and easier to reproduce [21,22]. These studies have also attempted to standardize normal values of PWV allocated into percentiles [23,24,25].

Considering that both methods are predisposed to errors, especially in the pediatric population, some researchers have attempted to develop a more precise modality to determine the distance covered by the pulse wave. In 2010, Jo et. al. [10] conducted a study on a sample of 80 children, seeking to find a method to measure the distance covered by the pulse wave. This value would be used to calculate the PWV. The researchers measured the length of the thoracic aorta using direct echocardiography and established a linear regression equation to calculate the length of the thoracic aorta using the patient’s height: thoracic aortic length (cm) = 1.7 (cm) + 0.1 × height (cm). This method is highly reliable with regard to its practical applicability, being easily reproducible in clinical practice [5].

The aim of this study is to assess if PWV could be an adequate marker of vascular remodeling in a pediatric population with CKD, along with technical aspects of PWV measurements in children.

## 2. Materials and Methods

This single-center prospective observational cohort study enrolled 42 consecutively pediatric patients referred to the Nephrology and Hemodialysis Department of “M. S. Curie” Clinical Emergency Hospital. Twenty-five patients were females (59.5%), and 17 males, between 9 and 18 years old, with end-stage CKD, all undergoing dialysis; the patients were evaluated between September 2014 and September 2020. The exclusion criteria were as follows: other secondary forms of hypertension, congenital cardiovascular diseases, history of aortic surgery, aortic valve pathology, and inflammatory large-vessel vasculitis. One patient was excluded, who had Takayasu arteritis.

The patients were evaluated using Philips ultrasound machine Philips CX50, Philips Affiniti, and Philips iE33 with S5-1, S8-3, and 17-5 transducers and having continuous ECG monitoring during the whole examination. During the examination, we measured both systolic and diastolic blood pressure, weight, and height for all the patients. Blood pressure measurements were performed on the right arm at the examination time by an automatic oscillometric method after 5 min of rest in a supine position.

The patients were diagnosed with arterial hypertension (stage 1 or 2), controlled or uncontrolled by treatment, based on the Clinical Guideline of American Academy of Pediatrics, considering age, sex, and height percentiles, using median blood pressure values during 24-h ambulatory monitoring [26]. Blood pressure ambulatory measurements were made using the oscillometric device, *BTL Cardio Point*-*ABPM*; 30 min intervals for daytime and 60 min intervals overnight were used for BP measurement.

**Figure 1:**
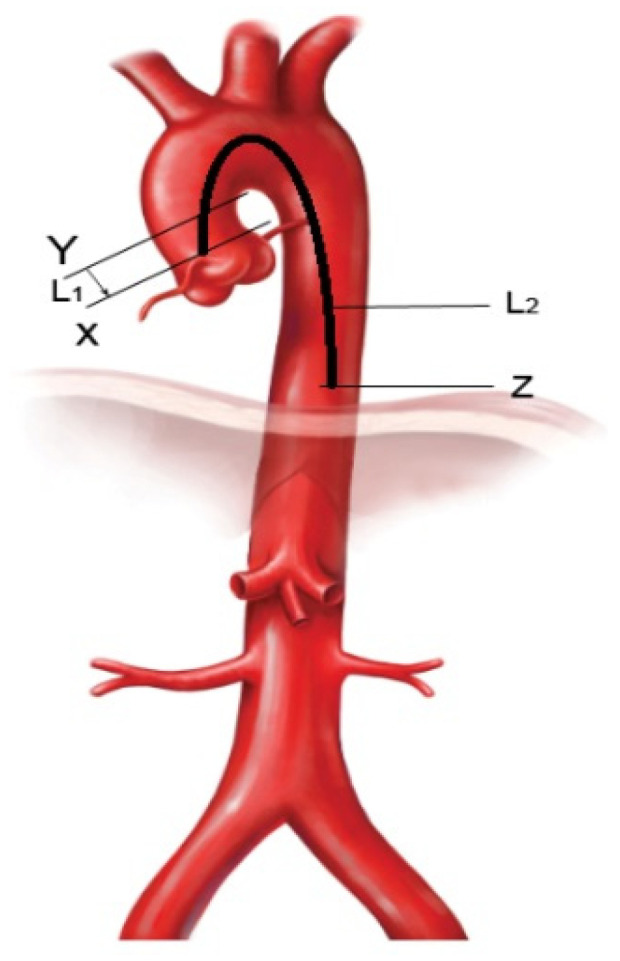
Schematical representation of the references used in the calculation of PWV (X—aortic valve level; Y—ascending aorta, 2 cm above the valve; Z—diaphragm level; L1 = distance from X to Y (2 cm), L2 = distance from X to Z (from the aortic valve to the diaphragm) = 1.7 cm + 0.1 × height (cm). PWV (ascending aorta) = L1/ΔT1, PWV (descending aorta) = L2/ΔT2.

**Figure 2:**
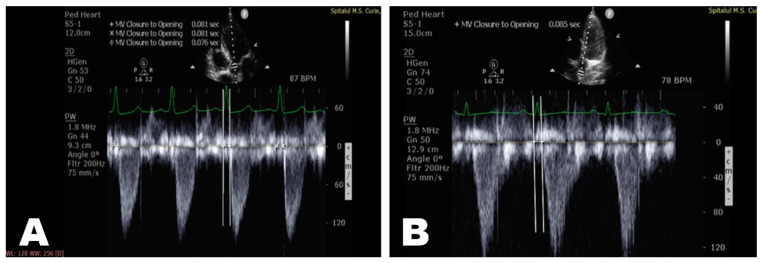
Measurement of ΔT1 (T1-T0 = 85 − 81 = 4 msec) in the ascending aorta (echocardiography—apical window 5C) (**A**,**B**).

PWV was measured non-invasively in the ascending aorta according to the formula L1/ΔT1, where L1 = 2 cm and ΔT1 represents the transit time of the pulse wave from the aortic valve (X point) to the established reference in the ascending aorta (Y point—2 cm from the aortic valve). Transit time was calculated as being the difference between the time from the start of the QRS complex to the beginning of the systolic wave at the Y point and the time from the start of the QRS complex to the beginning of the systolic flow at the X point (Figure 1 and Figure 2).

**Figure 3:**
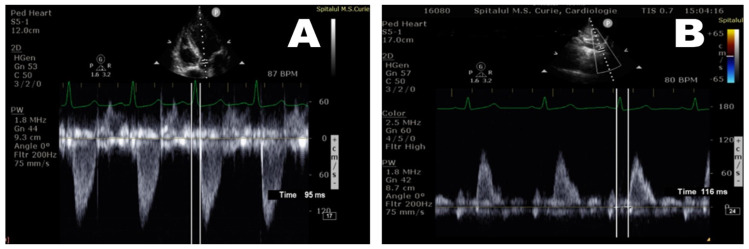
Measurement of ΔT2 (T2-T1 = 116 − 95 = 21 ms) in the descending aorta—echocardiography 5C apical window (**A**) and subcostal view (**B**).

To minimize the errors, the time used for the final calculation represented an average of five consecutive measurements. In the DescAo, we measured PWV according to the formula L2/ΔT2, where L2 represents the length of the aorta from the aortic valve to the diaphragm (X-Z distance—Figure 1), and ΔT2 stands for the transit time of the pulse wave travelling from the aortic valve (X point) to the reference in the DescAo (Z point—diaphragm level). Transit time was calculated as being the difference between the time measured from the start of the QRS complex to the beginning of the systolic wave in the Z point, and the time from the start of QRS to the beginning of the systolic flow in the X point (Figure 1 and Figure 3).

Given the difficulty of measuring the aortic length (L2), we conducted a literature review upon this subject and concluded that the method that would be least prone to errors is according to the following formula [9]: L2 = length of the thoracic aorta (from the aortic valve to diaphragm) = 1.7 cm + 0.1 × height (cm).

PWV values were compared to the normal values allocated to age percentiles by gender of PWV in the pediatric population [22,23,24]; vascular stiffness (with diminished vascular elasticity) was considered as being the presence of a PWV over or equal to the value on the 95th percentile. We prefer to use for comparison the normal values adapted by age, not by height, to avoid possible errors given by reduced height for the age in these patients (comparison with normal values for their height would lead to lower specificity of the method and overestimation of the presence of vascular stiffness given the fact that normal values increase with age). The presence of left ventricular hypertrophy and cardiac remodeling was objectively determined based on the left ventricular mass index and the left ventricular relative wall thickness.

The calculation of the left ventricle index was done according to the formula approved by the Echocardiography Societies, and we used the software implemented on the http://www.csecho.ca/wp-content/themes/twentyeleven-csecho/cardiomath (accessed on 24 December 2021), platform.

The left ventricular mass (LVM) was calculated according to a previously published methodology [18] on basis of end-diastolic LV diameter and wall thickness.

LVM (g) = 0.8{1.04[([LVEDD + IVSd + PWd]^3^ − LVEDD^3^)]} + 0.6 [27], where 1.04 = specific gravity of the myocardium (g/cm3), LVEDD is left ventricular end-diastolic diameter, IVSd is interventricular septal thickness at end-diastole (mm), and PWd is posterior wall thickness at end-diastole (mm).

Relative wall thickness (RWT) allows for further classification of increased LV mass as either concentric hypertrophy (RWT > 0.42) or eccentric hypertrophy (RWT ≤ 0.42).

RWT = 2 × PWd/LVEDD

**Figure 4:**
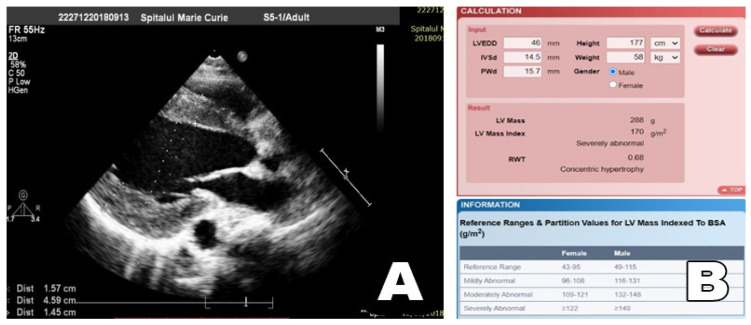
(**A**) Measurement of the LV wall thickness by 2D echocardiography, parasternal long-axis view (14-year-old patient from the study sample); (**B**)—calculation of the LVMI for the same patient (LVMI = 170 g/m2—severe concentric LVH).

LVMI (g/m2) was calculated as follows: LVMI = left ventricular mass/body surface area (BSA) [18,27]. (See Figure 4A,B).

### Data Analysis and Statistics

We performed the statistical analysis and created the charts using the software GraphPad Prism 9.2.0, USA; SPSS 27 (IBM SPSS, Chicago, IL, USA) and Analyse IT 5.5 (Microsoft Office Excel Add-on, Leeds, UK). The results were analyzed with the software Medcalc (Medcalc software bvba, version 11.5.1.0). Since most results were not normally distributed, all results are presented with their median and interquartile range (IQR). Differences between groups were assessed by t-test, or chi-squared test when appropriate. We compared the PWV values between the group with and without vascular stiffness, using a t-test. If the *p*-value of the predictor candidate in the univariate analysis was below 0.05, this predictor was included in the multivariable regression model.

## 3. Results

Anthropometric, clinical, and echocardiographic data of the patients included in the study group are presented below (See Table 1).

### Presence of Vascular Stiffness

Of the 42 patients from the study group, 32 patients (76.2%) had cardiac disease expressed by concentric remodeling (14.29%), concentric LVH (40.48%), or eccentric LVH (21.43%), and 22 were females. Fifteen (46.9%) patients had increased PWV, LV hypertrophy/remodeling was present in all cases, and 17 patients had LV hypertrophy/remodeling without increased PWV.

The characteristics of the patients and the values of the results obtained by echocardiography of the patients from the study group are presented in Table 2.

The mean PWV of the 15 children with LVH and vascular stiffness was 4.53 m/sec in AscAo and 5.279 m/sec in DescAo.

Linear regression was used to determine the echocardiography parameters among the group without LVH and the groups with LVH +/- vascular stiffness. There is a positive correlation with statistical significance (*p* < 0.05) in the group with LVH and vascular stiffness for PWV in DescAo, and age, height, SBP and DBP, and type of RRT. All patients with increased PWV were hypertensive (versus 70.6% hypertensive patients among patients without vascular stiffness)—See Table 2. The presence of vascular stiffness in AscAo does not seem to correlate with age, SBP, DBP, period of dialysis, or type of renal replacement therapy (*p* > 0.1). Values of the individual correlations are shown in Table 3.

In our cohort, 25 of 42 patients were females (95% CI = 0.44–0.73). In the group with cardiac impairment, 21 of 32 patients were females (95% CI = 0.48–0.79), and in the group with vascular impairment associated with cardiac disease, we found 12 females (95% CI = 0.54–0.93) of 15 patients.

**Figure 5:**
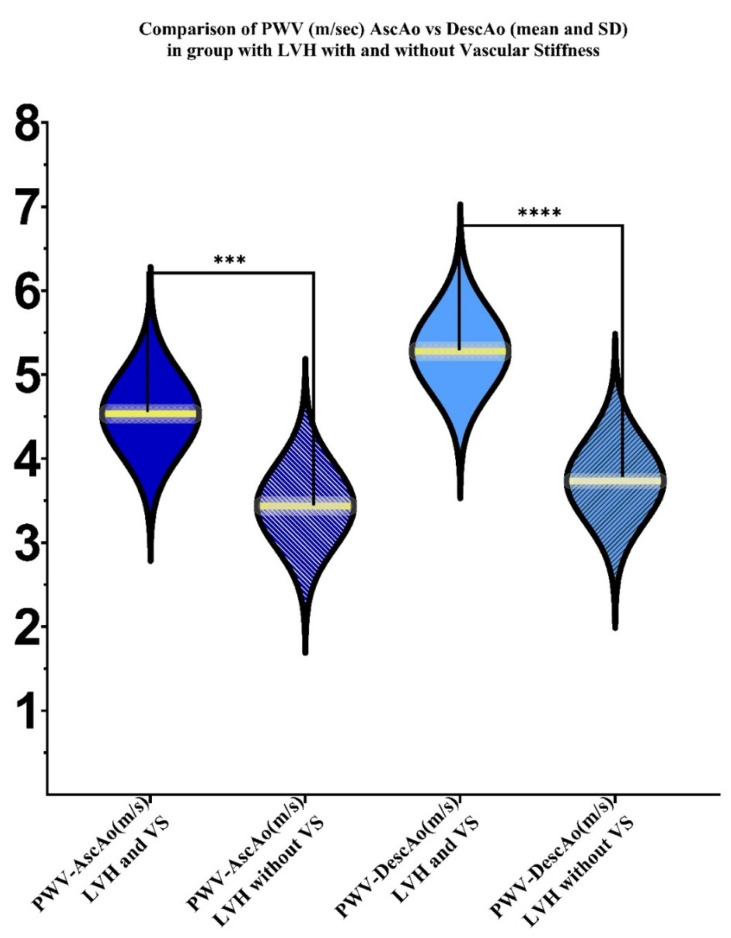
Violin plot of PWV values in the AscAo and DescAo, in groups with LVH with vascular stiffness (VS) and without vascular stiffness, by two-way ANOVA. *p* < 0.001, (***), *p* < 0.0001 (****).

Comparison of AscAo and DescAo PWV values between groups with LVH and vascular stiffness (VS) and those with LVH but without vascular stiffness, revealed statistically significant difference in both AscAo and DescAo (*p* value < 0.0001). We observed higher values in group with LVH and arterial stiffness, in PWV AscAo (mean = 4.533 m/sec, 95% CI [4.077–4.990 m/sec], *p* < 0.001), than in the group without arterial stiffness (mean = 3.650 m/sec, [3.386–3.914 m/sec], *p* < 0.001). Similar findings were observed as we compared the DescAo PWV in the group with vascular stiffness (mean = 5.24 m/s, (3.46–6.92 m/sec), *p* < 0.001), and with the non-vascular stiffness group (mean = 3.795 m/sec, (3.380–4.210 m/sec), *p* < 0.001) (Figure 5).

**Figure 6:**
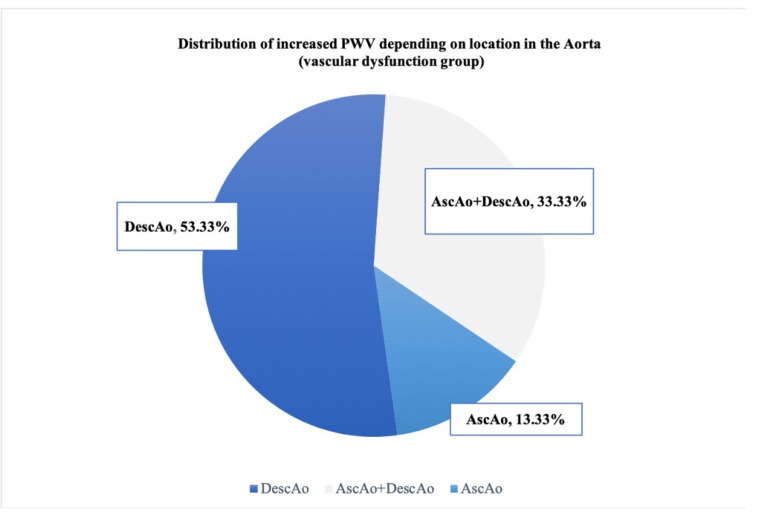
Distribution of increased PWV depending on location in the aorta (vascular dysfunction group).

Among the group of 15 participants with vascular stiffness, only seven patients had increased PWV in the AscAo with or without concomitant increased PWV in the DescAo. Higher PWV levels were found in the DescAo in 13/15 patients who had PWV > 95th percentile (including one patient with high PWV in both AscAo and DescAo). Five out of fifteen patients showed an increase in PWV in both the AscAo and the DescAo. Eight out of fifteen had increased PWV in the DescAo, and two out of fifteen had increased PWV in the AscAo (Figure 6).

**Figure 7:**
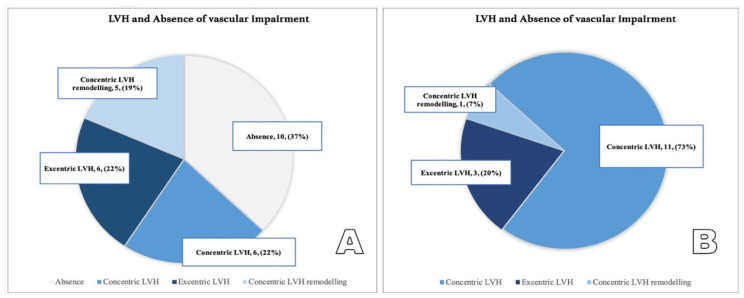
(**A**,**B**) Correlation between LVH type in patients without vascular impairment (**A**) and patients with vascular impairment (**B**).

**Figure 8:**
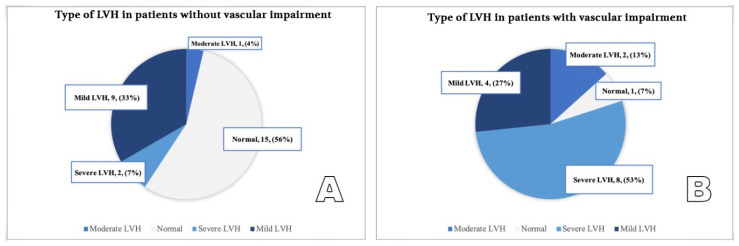
(**A**,**B**) Severity of LVH in patients without vascular impairment (**A**) compared to patients with vascular impairment (**B**).

The presence of uncontrolled hypertension is positively correlated with vascular stiffness (*p* < 0.01): 60% of those with increased PWV in the aorta present with untreated/insufficiently treated hypertension (versus 17.64% among patients without vascular dysfunction). Additionally, among patients with vascular stiffness, patients with concentric LVH and patients with severe LVH are predominant (Table 2, Figure 7 and Figure 8).

The occurrence of vascular dysfunction in the AscAo was statistically related to the severity of arterial hypertension—all seven patients with ascending aorta damage had stage 2 hypertension. However, descending aorta damage was not correlated with the severity of hypertension—half of the patients with isolated DescAo dysfunction presented only stage 1 hypertension. Vascular damage of the AscAo was not related to a longer period of dialysis, compared with patients with dysfunction of the DescAo (see Table 3).

## 4. Discussion

This study shows that vascular stiffness in children with CKD based on increased PWV is significantly correlated with the presence of cardiac impairment, defined as the presence of LV concentric remodeling or hypertrophy.

Given that all patients who had elevated PWV values (above the 95th percentile of normal age values) were found to have these echocardiographic myocardial findings versus 63% of those without vascular stiffness, the implications of PWV for cardiovascular risk estimation require confirmation. Elevated PWV values indicate increased arterial stiffness and may represent an important parameter for identifying children with CKD and children at high risk for major cardiovascular events (stroke, myocardial infarction, and cardiovascular death).

Our findings raise the following questions:

(1) Does impairment of large vessels elasticity occur later in the course of CKD than apparition of abnormal myocardial findings?
(2) Is the PWV cut-off value set at the 95th percentile too high and does it require adjustment to a lower threshold to increase the sensitivity of the method for determining vascular stiffness utilizing this noninvasive modality?

To answer these questions, further research is required to correlate PWV values with other manifestations of abnormal vascular elasticity, such as aortic distensibility or intima-media thickness at the carotid.

Another finding we would like to emphasize is the higher incidence of descending aorta stiffness in patients with CKD in comparison to the ascending aorta. We suspect that this finding is related to proximal/distal structural aortic wall differences, which in turn may be related to the distal to the proximal progression of biomechanical changes, similar to the damage described in the aorta with advancing age in the adult population [25]. This pathological progression is consistent with our findings. Specifically, the appearance of the initial damage at the level of small peripheral systemic vessels, leading to systemic hypertension and LVH and subsequently to large arteries disease from distality to proximal segments [26].

In the core of our work is the technique we utilized to measure PWV. The most important advantage of the PWV calculation method we utilized is the ability to measure PWV across a short segment of the ascending aorta. This measurement cannot be accomplished using the digital subtraction method, which is much more frequently used, especially in the adult population. This advantage allowed for the demonstration of the different characteristics of the ascending versus descending aorta PWV. Other advantages offered by this technique are the accuracy of measurements of the pulse wave transit times at the level of the descending aorta, as well as low costs, requiring only an ultrasound machine with adequate software and an experienced sonographer.

Based on our literature search, comparison of the aortic stiffness at different levels of the aorta in children with CKD has not been the focus of other research studies so far. Studies conducted since 2000 have confirmed, similar to our study, the increase in PWV values in these patients [10,11,12,13,14]. The association of increased PWV with systemic hypertension and left ventricular hypertrophy has been studied in adults with chronic end-stage renal disease but not in pediatrics. A study by Nitta et al. published in 2020 revealed a positive correlation between PWV values and LV mass index in the dialysis adult population [25]. As for pediatric studies, Sinha and colleagues (2015) and Savand and colleagues (2017) showed an association between vascular stiffness and hypertension but no link to the degree of impaired renal function [26,27]. A 2020 study conducted by Raina and colleagues concluded that the level of blood pressure and vascular stiffness index was associated with LV mass index and decrease in GFR, similar to that in the adult population; however, that study does not use PWV as a marker for vascular stiffness [28].

Although our study provides useful information about the association of PWV as a marker of vascular stiffness and LV hypertrophy or ventricular remodeling in pediatric patients, it is important to consider several limitations. First, our study population included significant gender differences that are hard to interpret and likely related to the overall small number of study patients. Second, our single center study design is prone to bias as well.

In 2009, the American Heart Association emphasized the importance of standardizing noninvasive methods for quantifying the cardiovascular risk in the pediatric population, mentioning the usefulness of PWV as a marker of end-organ damage [29]. Unfortunately, the measurement of PWV in children is problematic because of challenges in the accurate measurement of the aortic length. Our approach allowed for the overcoming of this challenge. However, because of the lack of standardization, larger studies are required to evaluate our findings.

Increased arterial stiffness was demonstrated to be the vascular hallmark of patients with end-stage renal disease independently of age, blood pressure, and standard risk factors [30]. Therapeutic approaches for normalization of arterial stiffness represent an important aim in the treatment of pediatric patients with CKD. Similar to adults, treatment strategies for children should be focused on the correction of traditional CV risk factors, including normalization of blood pressure, medication to reduce lipid levels, and correction of volume in dialyzed patients. Further longitudinal, randomized, and controlled studies are required to clearly prove that vascular dysfunction in children with CKD is a strong predictor of cardiovascular events (similar to adults) and to search if a decrease of arterial stiffness in children results in a lower incidence of cardiovascular disease later in life.

The limitations of this study include the small size of the cohort, single-center study, and significant difference in the gender distribution. Other study limitations include the absence of a normal control group to compare with.

## 5. Conclusions

Elevated PWV values indicate increased arterial stiffness and may represent an important parameter for identifying children with CKD and children at high risk for major cardiovascular events (stroke, myocardial infarction, and cardiovascular death). The technique used for the assessment of PWV value could be based only on echocardiographic measurements (as we did in our study), but it requires a skilled sonographer to lower possible measurements errors.

Further studies, including larger numbers of patients, are necessary to conclude if the cut-off value used in our study for the diagnosis of vascular dysfunction (95th percentile) has the best sensitivity and specificity for detection of this pathology.

The non-invasive technique for PWV is an important issue and require further refinement for it to become an important part of the clinical routine. Future studies should provide robust evidence of increased PWV in either the AscAo or the DescAo, in children with HD who had LV structural abnormalities.

## Figures and Tables

**Table 1:** Anthropometric, clinical, and echocardiographic data in the patients included in the study group, in children without LVH and group with LVH (with vascular stiffness and without vascular stiffness). Abbreviations: ** *p* ≤ 0.01, *** *p* ≤ 0.001. Data are expressed as (mean ± SD and 95% confidence interval) and *n*-number of patients (percentage).

Parameter	Total Group (*n* = 42)	Without LVH	LVH Group	
		*n* = 10	Vascular Stiffness Absent (*n* = 17)	Vascular Stiffness Present (*n* = 15)
Gender: *n* = female, male (%female) **	25,17 (59.5)	4,6 (40)	13,14 (48.15)	12,3 (80)
Age (years)	14 [11.9; 17]	14.1 [12.75;15.5]	14.2 [11; 17]	13.5 [11; 17]
Type of renal replacement therapy (RRT)				
HD -*n* (%)	39 (92.9)	9 (90)	15 (88.2)	15 (100)
PD -*n* (%)	3 (7.1)	1 (10)	2 (11.76)	0 (0)
AHT (stage) **				
Arterial hypertension stage I-*n* (%)	18 (42.9)	5 (50)	9 (52.9)	4(21.7)
Arterial hypertension stage II -*n* (%)	14 (33.3)	0	3 (17.65)	11(73.3)
Absent (%)	10 (23.8)	5 (50)	5 (29.4)	0
Controlled BP-*n* (%) **	30 (71.43)	10 (100)	14 (82.35)	6 (40)
T0(QRS—AoV) (msec)	70 [63.9;80.1]	65.7 [63.75;72]	74.47 [60;92]	72 [64;80]
T1(QRS—AscAo) (msec)	75 [67;84]	70.8 [69.75;75.25]	80.18 [66;97.5]	76.6 [67;84]
T2(QRS –DescAo) (msec)	109 [98.9;117.1]	107.3 [104.3;115]	118.8 [101;136]	104 [92;113]
PWV—AscAo (m/sec) **	4 [3.3;4]	3.65 [3.3;4]	3.44 [3.3; 4]	4.53 [4; 6.92]
95th percentile— PWV Asc Aorta -*n* (%)	7 (16.7)	0	0	7 (46.67)
PWV—DescAo (m/sec) ***	4.05 [3.61;4.75]	3.795 [3.58;4.39]	3.73 [3.46;4.015]	5.279 [4.64;5.83]
95th percentile—PWV DescAo -*n* (%)	13 (31)	0	0	13 (86.67)
LVH grade				
normal	16 (38.1)	0	15(88.24)	1 (6.67)
Mild -*n* (%)	13 (31)	0	9 (52.94)	4 (26.67)
Moderate -*n* (%)	3 (7.14)	0	1 (5.882)	2 (13.33)
Severe -*n* (%)	10 (23.8)	0	2 (11.765)	8 (53.33)
LVH type **				
Concentric remodeling-*n* (%)	6 (14.29)	0	5 (29.41)	1 (6.67)
Eccentric LVH-*n* (%)	9 (21.43)	0	6 (35.29)	3 (20)
Concentric LVH -*n* (%)	17 (40.48)	0	6 (35.29)	11 (73.33)

**Table 2:** Patient’s characteristics and echocardiographic measurements (mean ± SD and 95% confidence interval).

Patient Characteristics	Without LVH	Concentric Remodeling	Concentric LVH	Eccentric LVH
N, total = 42	10 (23.8%)	6 (14.29%)	17 (40.48%)	9 (21.43%)
Gender (N), M/F	6/4	3/3	6/11	5/4
Age (years)	14.10 ± 2.025 (12.75–15.50)	14.17 ± 2.137 (12.75–15.75)	13.47 ± 3.281 (10.5–17)	15.25 ± 3.151 (12.25–18)
E (cm/s) (Peak velocity of early diastolic transmitral flow)	104.9 ± 11.11 (95–113.3)	106.3 ± 12.96 (95–120.5)	105.8 ± 28.84 (87.5–115.5)	97.63 ± 10.11 (88.75–104.5)
E’ cm/s (Peak velocity of early diastolic mitral annular motion as determined by pulsed wave Doppler)	16.06 ± 1.757 (15.08–17)	16.32 ± 1.07 (15.08–17)	13.68 ± 3.121 (11.3–16.6)	13.83 ± 3.844 (9.475–17)
E/E’ (Ratio of E to E’)	6.663 ± 1.389 (5.6–7.993)	6.585 ± 1.23 (5.6–7.993)	8.098 ± 3.046 (5.75–8.89)	7.844 ± 2.746 (5.735–11.16)
Isovolumic relaxation time (IRVT)msec	63.6 ± 15.71 (44.5–77)	64.17 ± 16.5 (44.5–80)	69.71 ± 15.77 (58.5–76.5)	67.88 ± 8.408 (60.75–74)
IVCT msec Isovolumic (isovolumetric) contraction time	53.6 ± 10.51 (50–57)	50.5 ± 9.138 (46.25–57)	75.12 ± 17.6 (57–88)	65.25 ± 14.34 (50.5–81.5)
LV(lateral)Sm cm/s Sm: systolic myocardial velocity.	10.17 ± 1.338 (9.150–12)	10.28 ± 1.372 (9.15–12)	10.17 ± 3.034 (7.975–11.25)	10.15 ± 1.425 (9.350–11.38)
MAPSE LAT (mm) Mitral annular plane systolic excursion (M-mode)	13.55 ± 1.165 (13–15)	13.42 ± 1.357 (12.63–15)	13.18 ± 2.351 (11–15.50)	13.16 ± 1.845 (11.78–13.9)
IVS (mm) interventricular sept	8.5 ± 1.202 (7.5–10)	8.667 ± 1.291 (8–10)	11.92 ± 1.9 (10.5–13.5)	10.25 ± 1.75 (9–10.88)
PW (mm) Posterior wall	7.7 ± 0.856 (7.5–8.25)	7.583 ± 0.97 (7.125–8.250)	11.39 ± 2.4 (9.85–12.75)	9.438 ± 1.613 (8.625–9.875)
Systolic blood pressure (SBP)	122 (115–132.5)	122.5 ± 9.354 (115–128.8)	137.1 ± 25.31 (117.5–160)	128.8 ± 22.8 (120–130)
Diastolic blood pressure (DBP)	77.5 (63.75–90)	75 ± 10.95 (63.75–82.5)	84.12 ± 20.71 (70–100)	79.75 ± 9.8 (74.75–80)
Left ventricle (LV) g/m^2^	87.6 ± 12.53 (76.75–95.75)	87.67 ± 14.15 (75.25–99)	150 ± 56.4 (111.5–166)	128.1 ± 44.92 (97.25–133.5)
LV (Diameter)-(mm)	42.1 ± 5.131 (41.5–44.5)	40.5 ± 5.857 (37.25–44)	42.21 ± 7 (36–48)	47.63 ± 3.335 (44.5–50.75)
LV(D) (mm) Z SCORE	0.049 ± 0.49 (−0.08–0.38)	-0.10 ± 0.6 (−0.615–0.29)	−0.07 ± 1.334 (−0.915–0.96)	0.97 ± 0.58 (0.34–1.528)
LV mass index g/m^2^	87.6 ± 12.53 (76.75–95.75)	87.67 ± 14.15 (75.25–99)	150 ± 56.41 (111.5–166)	128 ± 44.92 (97.25–133.5)
LVEF, % (left ventricular ejection fraction)	62 ± 4.216 (60–62.5)	63.33 ± 5.164 (60–70)	58.82 ± 10.08 (55–65)	57.88 ± 6.896 (51.25–65)

**Table 3:** Pearson’s correlation coefficients for PWV in the groups without LVH, with LVH with vascular stiffness, and LWH without vascular stiffness. Abbreviations: SBP-systolic blood pressure, DBP—diastolic blood pressure, and RRT-renal replacement therapy.

Parameters	Group without LVH		Group with LVH and Vascular Stiffness		Group with LVH without Vascular Stiffness	
number patients, [%]	10, [23.8]		15 [35.71]		17 [40.48]	
PWV	PWV AscAo (m/s)	PWV DescAo (m/s)	PWV AscAo (m/s)	PWV DescAo (m/s)	PWV AscAo (m/s)	PWV DescAo (m/s)
Age	0.1	0.3	0.9	<0.0001	0.9619	0.5
Height	0.125	0.23	0.223	<0.05	0.06	0.6245
Weight	0.2	0.06	0.456	0.5	0.65	0.876
SBP	0.3	0.45	0.5	0.05	0.5	0.9
DBP	0.9140	0.1795	0.1	<0.0001	0.17	0.22
Type of RRT	0.45	0.125	*p* > 0.1	<0.0001	0.07	0.17
Period of dialysis	0.3	0.057	0.15	0.17	0.3	0.635

## Data Availability

Not applicable.
